# The 4G/5G polymorphism of plasminogen activator inhibitor type 1 is a predictor of allergic cough

**DOI:** 10.3389/fgene.2023.1139813

**Published:** 2023-02-24

**Authors:** Rui Tang, Xiaohong Lyu, Hong Li, Jinlyu Sun

**Affiliations:** ^1^ Allergy Department, State Key Laboratory of Complex Severe and Rare Diseases, Peking Union Medical College Hospital, Chinese Academy of Medical Sciences and Peking Union Medical College, Beijing, China; ^2^ Allergy Department, Beijing Key Laboratory of Precision Medicine for Diagnosis and Treatment of Allergic Diseases, National Clinical Research Center for Dermatologic and Immunologic Diseases, Peking Union Medical College Hospital, Chinese Academy of Medical Sciences and Peking Union Medical College, Beijing, China; ^3^ Eight-Year Program of Clinical Medicine, Peking Union Medical College, Chinese Academy of Medical Sciences, Beijing, China

**Keywords:** allergy and immunology, gene polymorphism, plasminogen activator inhibitor 1, cough, Hypersensitivity

## Abstract

**Background:** It has been suggested that genetic factors may be substantially linked to allergy disorders. This study aims to investigate the relationship between the genetic susceptibility of Chinese patients with allergy disorders and the polymorphisms of plasminogen activator inhibitor 1 gene (*PAI-1*) rs1799762, cysteinyl leukotriene receptor 1 gene (*CYSLTR1*) rs320995, gasdermin B gene (*GSDMB*) rs7216389, glycoprotein IIIa gene (*GPIIIa*) rs5918, glycoprotein Ib alpha gene (*GP1BA*) rs6065, platelet endothelial aggregation receptor 1 gene (*PEAR1*) rs12041331, and tumor necrosis factor alpha gene (*TNF-α*) rs1800629.

**Methods:** From the Peking Union Medical College Hospital, this study enrolled 60 healthy participants and 286 participants with allergic diseases. TaqMan-minor groove binder (MGB) quantitative polymerase chain reaction (qPCR) was used to examine the gene polymorphisms in each group.

**Results:** The TaqMan-MGB qPCR results were completely consistent with the DNA sequencing results, according to other studies in this medical center (Kappa = 1, *p* < .001). Only the distribution of PAI-1 rs1799762 was different between patients with allergic cough and healthy people (χ2 = 7.48, *p* = .0238). With regard to cough patients, the 4G4G and 5G5G genotypes were more frequent (allergic cough vs. healthy individuals: 4G4G 57.9% vs. 26.7%; 5G5G 20.0% vs. 13.3%), but the 4G5G genotype was more frequent in healthy people (allergic cough vs. healthy individuals: 45.7% vs. 60.0%). The *CYSLTR1* rs320995, *GSDMB* rs7216389, *GPIII*a rs5918, *GP1BA* rs6065, *PEAR1* rs12041331, and *TNF-α* rs1800629 polymorphisms, however, did not show any of such relationships.

**Conclusion:** The *PAI-1* rs1799762 polymorphisms may be associated with the genetic susceptibility of Chinese allergic disease patients with cough performance.

## 1 Introduction

Due to a recent spike in prevalence, allergic disorders have become a considerable social burden (Simon, 2019). The prevalence of allergic diseases in children ranges from 7% to 10% for asthma, 15%–20% for allergic rhinitis and conjunctivitis, and 15%–20% for atopic dermatitis worldwide (Eigenmann, 2005). These can have an impact on a patient’s quality of life and increase healthcare costs. The prevalence of allergy illnesses has emerged as one of the major issues facing China’s healthcare system as a result of the country’s enormous population. Asthma, allergic and non-allergic rhinitis, and food allergies can all be diagnosed and treated thanks to the molecular and cellular basis of fundamental and clinical immunology research, which has considerably enhanced our understanding of allergic illnesses in the last 10 years (Han et al., 2020).

Allergy and associated illnesses are strongly heritable. Genetic vulnerability to allergy illnesses may be considerably influenced by disease-related single nucleotide polymorphisms (SNPs), according to genome-wide association studies (GWAS) (Tamari and Hirota, 2014; Bønnelykke et al., 2015; Haider et al., 2022). Studies of monogenic disorders have uncovered important cellular processes and protein roles in allergies. These complementary techniques suggest genetic underpinnings for T helper 2 cell (Th2) immunity, T-cell differentiation, transforming growth factor beta (TGFβ) signaling, regulatory T-cell activity, and skin/mucosal function, as well as as-yet-unknown mechanisms linked to newly discovered genes (Bønnelykke et al., 2015).

As a result, it may be sensible to find SNPs that are crucial to the development of the allergic condition. Numerous potential genes were grabbing the attention of researchers among the pathophysiological mediators involved in allergic rhinitis, asthma, severe anaphylactic reactions, atopic dermatitis, and other allergic illnesses. Cysteiny1 leukotrienes (CYSLTs) have been suggested to aid in promoting bronchoconstriction, vascular hypermeability, and mucous hypersecretion (Zhang et al., 2006). The CYSLTs receptor cysteiny1 leukotriene receptor 1 (CYSLTR1) has become a key target for the treatment of asthma and rhinitis. Antagonists against CYSLTR1, including montelukast, pranlukast and zafirlukast, have been widely prescribed in clinical practices (Trinh et al., 2019). There was also research reporting that polymorphism of rs321029 on CYSLTR1 gene was not related to the susceptibility and severity of allergic rhinitis in children, but it is closely related with the efficacy of montelukast on allergic rhinitis (Zhao et al., 2021). Additionally, it was observed that persistent mucosal inflammation may be associated to gasdermin B (GSDMB) (Moffatt et al., 2007). Moderate evidence exists for associations of the GSDMB rs7216389 variants with asthma (Zhao et al., 2015). GSDMB is a functional gene for both asthma susceptibility and severity. Single nucleotide polymorphisms in GSDMB associated with asthma severity, exacerbations, and GSDMB expression levels (Li et al., 2021).

The integrin, beta 3 (ITGB3) gene codes for glycoprotein IIIa (GPIIIa), the beta subunit of the platelet membrane adhesive protein receptor complex, which is a surface protein present in many tissues and involved in cell adhesion and cell-surface mediated signaling (Bennett, 2005). The leucine/proline polymorphism at position 33 (PlA1/A2) of the polypeptide chain correlates to the common ITGB3 polymorphism at codon 33 of exon 2 (rs5918). This SNP has been implicated as an asthma risk factor (Zimrin et al., 1990; Mikkelsson et al., 2000). The blocking of GPIIIa receptor with tirofiban can attenuate airway hyperresponsiveness and airway inflammation through the inhibition of platelet-eosinophil aggregation and activation (Kim et al., 2021).

The glycoprotein Ib alpha gene (*GP1BA*), which codes for the complex’s GP1ba transmembrane subunit, carries the SNPs rs2243093 and rs6065 (also known as the human platelet antigen-2, which leads to a threonine to methionine substitution at position 145). In an earlier investigation, GP1ba was linked to allergic airway disorders, especially severe, treatment-refractory asthma (Wu et al., 2021). Cardiovascular illness was found to be highly correlated with the SNPs of platelet endothelial aggregation receptor 1 (PEAR1), particularly rs12041331 and rs12566888 (Pi et al., 2019). The unique relationship between IgE-mediated allergy and cardiovascular disease was discovered to be PEAR1 (Sun et al., 2015). The omalizumab might be able to relieve the IgE-mediated inhibition of the FcεR1α-PEAR1 interaction, suggesting that omalizumab treatment could lead to alterations in the regulation of PEAR1 signaling (Sun et al., 2015).

The fibrinolytic system is significantly inhibited by plasminogen activator inhibitor 1 (PAI-1). According to recent research, PAI-1 controls airway remodeling, hyperresponsiveness, and allergic inflammation, which may contribute to the onset of asthma (Ma et al., 2009). There was a report showing that PAI-1 as a marker of esophageal functional changed in pediatric eosinophilic esophagitis (Williamson et al., 2022). A meta-analysis suggested that the −675 4G/5G polymorphism of PAI-1 gene was a risk factor of asthma (Nie et al., 2012). In a latest report, tumor necrosis factor alpha gene (*TNF-α*)*-308G/A* polymorphism in asthma patients was found to be associated with their metabolic profile (Yang et al., 2015). A large-scale meta-analysis supports a strong association between the TNF-α gene promoter polymorphism (−308G/A) and the development to asthma in both children and adults (Kang et al., 2019).

The association between allergy disorders and SNPs in the *CYSLTR1* rs320995, *GSDMB* rs7216389, *GPIII*a rs5918, *GP1BA* rs6065, *PEAR1* rs12041331, *PAI-1* rs1799762, and *TNF-α* rs1800629 gene has, regrettably, only been the subject of a few reports. As a result, it is a subject worth researching and may be applied to clinical detection.

The purpose of this study is to determine if gene polymorphisms in the *CYSLTR1* rs320995, *GSDMB* rs7216389, *GPIII*a rs5918, *GP1BA* rs6065, *PEAR1* rs12041331, *PAI-1* rs1799762, and *TNF-α* rs1800629 gene are related to various allergy diseases in Chinese patients.

## 2 Materials and methods

### 2.1 Ethics

The 1964 Helsinki declaration and its later amendments or analogous ethical standards were followed in all procedures carried out in studies involving human participants, as determined by the institutional and/or national research committee. The Chinese Academy of Medical Sciences and Peking Union Medical College Hospital Drug Clinical Trial Ethics Committee gave their clearance for this study, registration information: No. 002062, ethics approval No. KS2019282.

### 2.2 Study design and participants

We carried out a prospective study at the Peking Union Medical College Hospital (PUMCH) in Beijing, China, between July 2019 and December 2019.

In accordance with the patients’ presentations and the outcomes of auxiliary tests, patients underwent normal diagnostic workups. Including criteria for allergy patients (Simon, 2019): diagnosed with allergy diseases by clinical doctors, including allergic rhinitis, asthma, urticaria, atopic dermatitis, cough, atopic conjunctivitis, eczema, or a history of severe anaphylactic reaction (Eigenmann, 2005); positive serum specific IgE, positive skin prick test or intradermal test. Excluding criteria for allergy patients (Simon, 2019): patients with serious other diseases, such as diabetes, liver disease, kidney disease, *etc.*, (Eigenmann, 2005); Immunocompromised patients.

Including criteria for healthy participants (Simon, 2019): No symptoms of any allergic diseases, including allergic rhinitis, allergic asthma, atopic dermatitis, allergic conjunctivitis, *etc.,* (Eigenmann, 2005) No history of allergic diseases, family history (Han et al., 2020). No other immune system diseases (Bønnelykke et al., 2015). No organic disease (Tamari and Hirota, 2014). Voluntary acceptance of disease-related questionnaires (Haider et al., 2022). No participation in any drug clinical trials within 3 months. Excluding criteria for healthy participants (Simon, 2019): history of allergic diseases or chronic medical conditions associated with allergy diseases in this study (Eigenmann, 2005); history of significant allergen exposure (Han et al., 2020); patients with serious other diseases, such as diabetes, liver disease, kidney disease, *etc.*, (Bønnelykke et al., 2015); Immunocompromised patients.

### 2.3 Clinical information collecting

After informed consent, we collected information on the patients’ plasma allergen sIgE levels, disease duration (years), blood eosinophil counts, and whether desensitization was used. The demographic information about the study’s participants is displayed in Table 1.

**TABLE 1:** The demographic characteristics of the participants in this study.

Group	Number of subjects	Age, median (IQR)	Gender		Duration of disease (years)	The allergen- sIgE (kU/L)	Eosinophils (*10^9^/L)	Desensitization treatment, n (%)
			Male	Female				
All allergic disease subjects	286	30 (13,40)	130	156	4 (2, 7)	12.6 (2.2, 43.1)	0.33 (0.17, 0.64)	50 (17.5%)
Allergic rhinitis	278	29 (13,39)	128	150	4 (2,7)	12.6 (2.4,43.6)	0.33 (0.17,0.64)	48 (17.3%)
Asthma	66	32 (16,46)	26	40	5 (3,9.5)	23.3 (4.3,47.4)	0.43 (0.15,0.83)	20 (30.3%)
Severe anaphylactic reactions	3	16 (8,48)	2	1	2 (0,/)	89.4 (5.2,/)	0.19 (0,/)	0 (0%)
Urticaria	14	35 (25,48)	6	8	3 (1.5,7)	6.0 (0.5,37.1)	0.15 (0.07,0.38)	1 (7.1%)
Atopic dermatitis	8	13 (7,36)	2	6	4 (0,7)	65.6 (11.5,100.0)	0.26 (0.17,0.50)	1 (12.5%)
Cough	19	32 (12,39)	9	10	2.5 (0.9,7.5)	22.9 (3.4,49.3)	0.26 (0.11,0.91)	3 (15.8%)
Atopic conjunctivitis	35	29 (9,34)	16	19	2.5 (0.9,4.0)	11.0 (2.3,51.0)	0.50 (0.27,0.89)	8 (22.9%)
Eczema	5	10 (7,52)	2	3	1.0 (0.8,5.5)	13.8 (2.8,78.7)	0.32 (0.21,1.47)	1 (20.0%)
Healthy individuals	60	34 (31, 39)	12	48	—	—	—	—

### 2.4 TaqMan-MGB qPCR method

With the use of DNA extraction kits from Tianlong Technology Co. LTD. in Xi’an, China, genomic DNA was extracted from peripheral blood sample. Using a gene polymorphisms RT-PCR detection kit, the *CYSLTR1* rs320995, *GSDMB* rs7216389, *GPIII*a rs5918, *GP1BA* rs6065, *PEAR1* rs12041331, *PAI-1* rs1799762, and *TNF-α* rs1800629 genes were genotyped (Wuhan Healthcare Biotechnology Co., Ltd., Wuhan, China). According to the Applied Biosystems methodology, genotyping was accomplished by TaqMan chemistry utilizing the real-time Prism 3730XL Sequence Detection System (ABI Inc. CA, United States). In our prior research, we have demonstrated the effectiveness of the TaqMan-MGB qPCR kit for the detection of gene polymorphisms (Wang et al., 2021; Jin et al., 2022; Wang et al., 2022). The Kappa test was used to examine the agreement between DNA sequencing and TaqMan-MGB qPCR, with a Kappa value of 1 and a *p*-value <.001.

### 2.5 Data statistics and analysis

Excel 2022 (Microsoft Inc., United States), SPSS 26.0 (SPSS Inc., Chicago, IL, United States), R Project (version 4.2.0), and RStudio (Open-Source Edition) software were all used to analyze the data. To determine if the frequency distribution of polymorphisms across genomes was representative, the Hardy-Weinberg equilibrium (HWE) test was utilized. To determine if there was a significant difference in SNP between the illness group and the healthy control group, the Wilcoxon or Chi-square test was utilized. The “pwr” package in RStudio was used to do a statistical power analysis. Statistics were judged significant at *p* < .05.

## 3 Results

### 3.1 Demographic characteristics of participants

Table 1 shows the demographic information about the study’s participants. With a median age of 30, the 286 patients with allergic diseases included 130 men and 156 women. There were 178 patients with allergic rhinitis, 66 with asthma, 3 with severe anaphylactic reactions, 14 with urticaria, 8 with atopic dermatitis, 19 with cough, 35 with atopic conjunctivitis, and 5 with eczema, according to their clinical performance. Additionally, 60 healthy adults with a median age of 34 and a gender split of 12 men and 48 women were as controls in the study.

The median duration of allergic diseases for all patients in this study was 4 (IQR, 2, 7) years. The median allergen-sIgE of all patients was 12.6 (IQR, 2.2, 43.1) kU/L. The median count of blood eosinophils in all patients was .33 (IQR, .17, .64)/L. And 17.5% allergy patients received desensitization treatment. Compared to other allergic diseases, more asthma patients received desensitization treatment (30.3%, *p* < .05).

**FIGURE 1:**
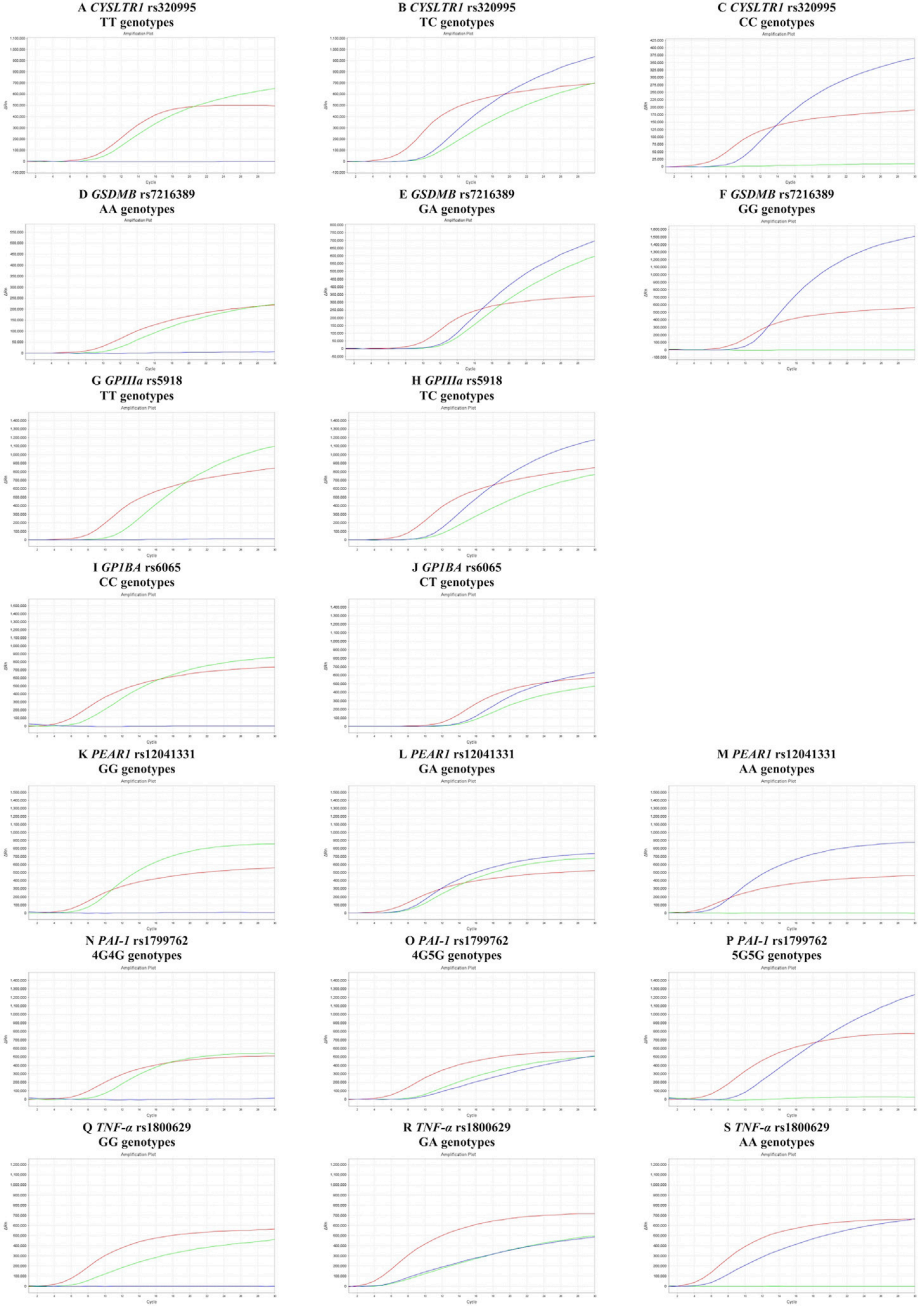
Amplification plots for genotypes of *CYSLTR1* rs320995, *GSDMB* rs7216389, *GPIIIa* rs5918, *GP1BA* rs6065, *PEAR1* rs12041331, *PAI-1* rs1799762 and *TNF-α* rs1800629. **(A–C)** amplification plots of *CYSLTR1* rs320995 TT, TC and CC genotypes in female. **(D–F)** amplification plots of *GSDMB* rs7216389 AA, GA, and GG genotypes. **(G,H)** amplification plots of *GPIIIa* rs5918 TT and TC genotypes. **(I,J)** amplification plots of *GP1BA* rs6065 CC and CT genotypes. **(K–M)** amplification plots of *PEAR1* rs12041331 GG, GA and AA genotypes. **(N–P)** amplification plots of *PAI-1* rs1799762 4G4G, 4G5G, and 5G5G genotypes. **(Q–S)** Amplification plots of *TNF-α* rs1800629 GG, GA and AA genotypes.

The amplification plots for genotypes of *CYSLTR1* rs320995, *GSDMB* rs7216389, *GPIII*a rs5918, *GP1BA* rs6065, *PEAR1* rs12041331, *PAI-1* rs1799762, and *TNF-α* rs1800629 are shown in Figure 1. The HWE equilibrium law was followed by the frequency distributions of the following polymorphisms in allergy patients: *CYSLTR1* rs320995, *GSDMB* rs7216389, *GPIII*a rs5918, *GP1BA* rs6065, *PEAR1* rs12041331, *PAI-1* rs1799762, and *TNF-α* rs1800629 (*p* > .05).

### 3.2 Analysis of association of *PAI-1* rs1799762 with genetic susceptibility to allergic disease

Only distribution of *PAI-1* rs1799762 differed from healthy controls in patients with allergic cough (χ2 = 7.48, *p* = .0238) (Table 2). The genotypes 4G4G and 5G5G were more common in cough patients (allergic cough vs. healthy individuals: 4G4G 57.9% vs. 26.7%; 5G5G 20.0% vs. 13.3%), but the 4G5G genotype was more common in healthy individuals (allergic cough vs. healthy individuals: 45.7% vs. 60.0%). Yet in other subgroups, no correlations between *PAI-1* rs1799762 and allergic rhinitis, asthma, urticaria, atopic dermatitis, atopic conjunctivitis, eczema, or severe anaphylactic reaction were found.

**TABLE 2:** Analysis of Association of PAI-1 rs1799762 (−675,4G5G) with Genetic Susceptibility to Diagnosis.

Diagnosis	Genotype	Group (%)		χ2	*P*
		Cases (*n* = 278)	Healthy individuals (*n* = 60)		
Allergic rhinitis	4G4G	93 (33.5)	16 (26.7)	1.72	0.4232
	4G5G	141 (50.7)	36 (60.0)		
	5G5G	44 (15.8)	8 (13.3)		
Asthma	4G4G	17 (25.8)	16 (26.7)	0.27	0.8737
	4G5G	38 (57.6)	36 (60.0)		
	5G5G	11 (16.7)	8 (13.3)		
Severe anaphylactic reactions	4G4G	1 (33.3)	16 (26.7)	1.19	0.5516
	4G5G	1 (33.3)	36 (60.0)		
	5G5G	1 (33.3)	8 (13.3)		
Urticaria	4G4G	5 (35.7)	16 (26.7)	1.42	0.4916
	4G5G	6 (42.9)	36 (60.0)		
	5G5G	3 (21.4)	8 (13.3)		
Atopic dermatitis	4G4G	2 (25.0)	16 (26.7)	0.02	0.9900
	4G5G	5 (62.5)	36 (60.0)		
	5G5G	1 (12.5)	8 (13.3)		
Cough	4G4G	11 (57.9)	16 (26.7)	7.48	0.0238
	4G5G	8 (42.1)	36 (60.0)		
	5G5G	0 (0.0)	8 (13.3)		
Atopic conjunctivitis	4G4G	12 (34.3)	16 (26.7)	1.88	0.3906
	4G5G	16 (45.7)	36 (60.0)		
	5G5G	7 (20.0)	8 (13.3)		
Eczema	4G4G	1 (20.0)	16 (26.7)	0.23	0.8914
	4G5G	3 (60.0)	36 (60.0)		
	5G5G	1 (20.0)	8 (13.3)		

### 3.3 Analysis of association of other gene with genetic susceptibility to allergic disease

The *CYSLTR1* rs320995, *GSDMB* rs7216389, *GPIII*a rs5918, *GP1BA* rs6065, *PEAR1* rs12041331, and *TNF-α* rs1800629 polymorphisms did not show any of such relationships (Table 3; Table 4; Table 5; Table 6; Table 7; Table 8).

**TABLE 3:** Analysis of Association of CYSLTR1 rs320995 with Genetic Susceptibility to Diagnosis.

Diagnosis	Genotype	Group (%)		χ2	*P*
		Cases (n = 278)	Healthy individuals (*n* = 60)		
Allergic rhinitis (Female)	TT	60 (40.0)	15 (31.3)	1.18	0.5543
	TC	82 (54.7)	30 (62.5)		
	CC	8 (5.3)	3 (6.25)		
Allergic rhinitis (Male)	T	91 (71.1)	9 (75.0)	0.08	0.7773
	C	37 (28.9)	3 (25.0)		
Asthma (Female)	TT	15 (37.5)	15 (31.3)	1.01	0.6035
	TC	21 (52.5)	30 (62.5)		
	CC	4 (10.0)	3 (6.25)		
Asthma (Male)	T	19 (73.1)	9 (75.0)	0.07	0.7913
	C	7 (26.9)	3 (25.0)		
Severe anaphylactic reactions (Female)	TT	1 (100.0)	15 (31.3)	2.11	0.3482
	TC	0 (0.0)	30 (62.5)		
	CC	0 (0.0)	3 (6.25)		
Severe anaphylactic reactions (Male)	T	1 (50.0)	9 (75.0)	0.01	0.9203
	C	1 (50.0)	3 (25.0)		
Urticaria (Female)	TT	1 (12.5)	15 (31.3)	1.99	0.3697
	TC	7 (87.5)	30 (62.5)		
	CC	0 (0.0)	3 (6.25)		
Urticaria (Male)	T	4 (66.7)	9 (75.0)	0.03	0.8625
	C	2 (33.3)	3 (25.0)		
Atopic dermatitis (Female)	TT	2 (33.3)	15 (31.3)	0.40	0.8187
	TC	4 (66.7)	30 (62.5)		
	CC	0 (0.0)	3 (6.25)		
Atopic dermatitis (Male)	T	2 (100.0)	9 (75.0)	0.02	0.8875
	C	0 (0.0)	3 (25.0)		
Cough (Female)	TT	4 (40.0)	15 (31.3)	0.83	0.6603
	TC	6 (60.0)	30 (62.5)		
	CC	0 (0.0)	3 (6.25)		
Cough (Male)	T	5 (55.6)	9 (75.0)	0.22	0.639
	C	4 (44.4)	3 (25.0)		
Atopic conjunctivitis (Female)	TT	8 (42.1)	15 (31.3)	0.71	0.7012
	TC	10 (52.6)	30 (62.5)		
	CC	1 (5.3)	3 (6.25)		
Atopic conjunctivitis (Male)	T	12 (75.0)	9 (75.0)	0.19	0.6629
	C	4 (25.0)	3 (25.0)		
Eczema (Female)	TT	2 (66.7)	15 (31.3)	1.65	0.4382
	TC	1 (33.3)	30 (62.5)		
	CC	0 (0.0)	3 (6.25)		
Eczema (Male)	T	1 (50.0)	9 (75.0)	0.01	0.9203
	C	1 (50.0)	3 (25.0)		

**TABLE 4:** Analysis of Association of GSDMB rs7216389 with Genetic Susceptibility to Diagnosis.

Diagnosis	Genotype	Group (%)		χ2	*P*
		Cases (*n* = 278)	Healthy individuals (*n* = 60)		
Allergic rhinitis	GG	23 (8.3)	9 (15.0)	2.77	0.2503
	GA	108 (38.8)	20 (33.3)		
	AA	147 (52.9)	31 (51.7)		
					
Asthma	GG	5 (7.6)	9 (15.0)	1.78	0.4107
	GA	23 (34.8)	20 (33.3)		
	AA	38 (57.6)	31 (51.7)		
					
Severe anaphylactic reactions	GG	1 (33.3)	9 (15.0)	0.79	0.6737
	GA	1 (33.3)	20 (33.3)		
	AA	1 (33.3)	31 (51.7)		
					
Urticaria	GG	0 (0.0)	9 (15.0)	2.97	0.2265
	GA	7 (50.0)	20 (33.3)		
	AA	7 (50.0)	31 (51.7)		
					
Atopic dermatitis	GG	1 (12.5)	9 (15.0)	0.34	0.8437
	GA	2 (25.0)	20 (33.3)		
	AA	5 (62.5)	31 (51.7)		
					
Cough	GG	3 (15.8)	9 (15.0)	0.11	0.9465
	GA	7 (36.8)	20 (33.3)		
	AA	9 (47.4)	31 (51.7)		
					
Atopic conjunctivitis	GG	4 (11.4)	9 (15.0)	2.16	0.3396
	GA	17 (48.6)	20 (33.3)		
	AA	14 (40.0)	31 (51.7)		
					
Eczema	GG	0 (0.0)	9 (15.0)	1.80	0.4066
	GA	3 (60.0)	20 (33.3)		
	AA	2 (40.0)	31 (51.7)		

**TABLE 5:** Analysis of Association of GPIIIa rs5918 (T176C) with Genetic Susceptibility to Diagnosis.

Diagnosis	Genotype	Group (%)		χ2	*P*
		Cases (*n* = 278)	Healthy individuals (*n* = 60)		
Allergic rhinitis	TC	7 (2.5)	1 (1.7)	0.01	0.9203
	TT	271 (97.5)	59 (98.3)		
Asthma	TC	3 (4.5)	1 (1.7)	0.17	0.6801
	TT	63 (95.5)	59 (98.3)		
Severe anaphylactic reactions	TC	0 (0.0)	1 (1.7)	0.05	0.8231
	TT	3 (100.0)	59 (98.3)		
Urticaria	TC	0 (0.0)	1 (1.7)	0.64	0.4237
	TT	14 (100.0)	59 (98.3)		
Atopic dermatitis	TC	1 (12.5)	1 (1.7)	0.35	0.5541
	TT	7 (87.5)	59 (98.3)		
Cough	TC	0 (0.0)	1 (1.7)	0.37	0.5430
	TT	19 (100.0)	59 (98.3)		
Atopic conjunctivitis	TC	0 (0.0)	1 (1.7)	0.08	0.7773
	TT	35 (100.0)	59 (98.3)		
Eczema	TC	0 (0.0)	1 (1.7)	2.56	0.1096
	TT	5 (100.0)	59 (98.3)		

**TABLE 6:** Analysis of Association of GP1BA rs6065 (C5792T) with Genetic Susceptibility to Diagnosis.

Diagnosis	Genotype	Group (%)		χ2	*P*
		Cases (*n* = 278)	Healthy individuals (*n* = 60)		
Allergic rhinitis	CC	245 (88.1)	54 (90.0)	0.04	0.8415
	CT	33 (11.9)	6 (10.0)		
Asthma	CC	60 (90.9)	54 (90.0)	0.02	0.8875
	CT	6 (9.1)	6 (10.0)		
Severe anaphylactic reactions	CC	3 (100.0)	54 (90.0)	0.19	0.6629
	CT	0 (0.0)	6 (10.0)		
Urticaria	CC	14 (100.0)	54 (90.0)	0.48	0.4884
	CT	0 (0.0)	6 (10.0)		
Atopic dermatitis	CC	7 (87.5)	54 (90.0)	0.16	0.6892
	CT	1 (12.5)	6 (10.0)		
Cough	CC	18 (94.7)	54 (90.0)	0.03	0.8625
	CT	1 (5.3)	6 (10.0)		
Atopic conjunctivitis	CC	29 (82.9)	54 (90.0)	0.48	0.4884
	CT	6 (17.1)	6 (10.0)		
Eczema	CC	5 (100.0)	54 (90.0)	0.55	0.4583
	CT	0 (0.0)	6 (10.0)		

**TABLE 7:** Analysis of Association of PEAR1 rs12041331 (G2266A) with Genetic Susceptibility to Diagnosis.

Diagnosis	Genotype	Group (%)		χ2	*P*
		Cases (*n* = 278)	Healthy individuals (*n* = 60)		
Allergic rhinitis	AA	46 (16.5)	8 (13.3)	0.64	0.7261
	GA	134 (48.2)	32 (53.3)		
	GG	98 (35.3)	20 (33.3)		
Asthma	AA	6 (9.1)	8 (13.3)	0.61	0.7371
	GA	38 (57.6)	32 (53.3)		
	GG	22 (33.3)	20 (33.3)		
Severe anaphylactic reactions	AA	0 (0.0)	8 (13.3)	0.49	0.7827
	GA	2 (66.7)	32 (53.3)		
	GG	1 (33.3)	20 (33.3)		
Urticaria	AA	4 (28.6)	8 (13.3)	3.04	0.2187
	GA	8 (57.1)	32 (53.3)		
	GG	2 (14.3)	20 (33.3)		
Atopic dermatitis	AA	2 (25.0)	8 (13.3)	2.34	0.3104
	GA	2 (25.0)	32 (53.3)		
	GG	4 (50.0)	20 (33.3)		
Cough	AA	4 (21.1)	8 (13.3)	0.98	0.6126
	GA	8 (42.1)	32 (53.3)		
	GG	7 (36.8)	20 (33.3)		
Atopic conjunctivitis	AA	5 (14.3)	8 (13.3)	0.20	0.9048
	GA	17 (48.6)	32 (53.3)		
	GG	13 (37.1)	20 (33.3)		
Eczema	AA	1 (20.0)	8 (13.3)	0.44	0.8025
	GA	3 (60.0)	32 (53.3)		
	GG	1 (20.0)	20 (33.3)		

**TABLE 8:** Analysis of Association of TNF-α rs1800629 (−308 G>A) with Genetic Susceptibility to Diagnosis.

Diagnosis	Genotype	Group (%)		χ2	*P*
		Cases (*n* = 278)	Healthy individuals (*n* = 60)		
Allergic rhinitis	AA	2 (0.7)	0 (0.0)	1.08	0.5827
	GA	33 (11.9)	5 (8.3)		
	GG	243 (87.4)	55 (91.7)		
Asthma	AA	0 (0.0)	0 (0.0)	2.61	0.2712
	GA	12 (18.2)	5 (8.3)		
	GG	54 (81.8)	55 (91.7)		
Severe anaphylactic reactions	AA	0 (0.0)	0 (0.0)	0.27	0.8737
	GA	0 (0.0)	5 (8.3)		
	GG	3 (100.0)	55 (91.7)		
Urticaria	AA	0 (0.0)	0 (0.0)	0.47	0.7906
	GA	2 (14.3)	5 (8.3)		
	GG	12 (85.7)	55 (91.7)		
Atopic dermatitis	AA	0 (0.0)	0 (0.0)	0.72	0.6977
	GA	0 (0.0)	5 (8.3)		
	GG	8 (100.0)	55 (91.7)		
Cough	AA	0 (0.0)	0 (0.0)	0.88	0.6440
	GA	3 (15.8)	5 (8.3)		
	GG	16 (84.2)	55 (91.7)		
Atopic conjunctivitis	AA	0 (0.0)	0 (0.0)	1.68	0.4317
	GA	6 (17.1)	5 (8.3)		
	GG	29 (82.9)	55 (91.7)		
Eczema	AA	0 (0.0)	0 (0.0)	0.45	0.7985
	GA	0 (0.0)	5 (8.3)		
	GG	5 (100.0)	55 (91.7)		

## 4 Discussion

Allergic disorders are frequent, hazardous, and occasionally fatal to patients. Growing research has emphasized how SNPs are linked to illness risk and prognosis, which is crucial for personalized medicine. Nevertheless, the field of allergic diseases had few corresponding reports. TaqMan-MGB qPCR offered a reliable and affordable method for investigating SNPs with allergy disorders (Wang et al., 2021; Jin et al., 2022; Wang et al., 2022). Using this technology, we initially discovered that patients with allergic cough had a different distribution of the *PAI-1* rs1799762 gene than healthy people.

A key inhibitor of the fibrinolytic system is PAI-1. According to recent research, PAI-1 controls airway remodeling, hyperresponsiveness, and allergic inflammation, which may contribute to the onset of asthma (Ma et al., 2009). Many research are looking into the relationship between PAI-1 4G/5G polymorphisms and a variety of disorders, such as thrombosis (Zhang et al., 2020a), systemic lupus erythematosus (Anaya-Macias et al., 2020), thyroid eye disease (Katko et al., 2021), metabolic syndrome (Zhang et al., 2020b) and many more. Previous research from our center has shown that the *PAI-1* 4G/5G polymorphism may be useful for determining the prognosis of Chinese patients with venous thromboembolism (Wang et al., 2022).

The −675 4G/5G polymorphism of the *PAI-1* gene was implicated as an asthma risk factor in a 2012 meta-analysis encompassing 1817 cases and 2327 controls (Nie et al., 2012). The 4G allele of the 4G/5G polymorphism in the *PAI-1* gene may be a risk factor for IgE-mediated asthma and allergy disorders, according to comparative research published in 2002 (Bucková et al., 2002). Patients with asthma who carry at least one 4G allele of PAI-1 gene are more likely to experience allergic rhinitis symptoms (Lampalo et al., 2017). Turkish children with asthma or allergic rhinitis had a higher prevalence of the *PAI-1* 4G variant than their healthy classmates, according to a Turkish study (Ozbek et al., 2009). Additionally, it was discovered that the genes *FCER1B* and *PAI-1* interact synergistically to increase asthma susceptibility (Hizawa et al., 2006).

The mechanism between asthma and *PAI-1* 4G/5G was explored by Ma et al. (2008). They discovered that upstream stimulatory factor-1’s binding to the E−4G/5G controls the production of PAI-1 in mast cells, a large source of PAI-1 and essential effector cells in asthma, in a manner, that is, reliant on the 4G/5G polymorphism. The primary genetic determinant of PAI-1 expression is the single guanosine nucleotide deletion/insertion polymorphism (4G/5G) at −675 base pairs of the *PAI-1* gene. People with the 4G/4G genotype have plasma PAI-1 levels that are higher than people with the 5G/5G genotype.

The 4G/5G polymorphism of *PAI-1* did not significantly differ between asthma patients and healthy individuals, according to this study. In contrast to healthy participants in this study, allergic patients with cough performance had higher levels of 4G4G and 5G5G. Additionally, a prior study found no statistically significant difference between the distribution of 4G/4G, 4G/5G, and 5G/5G between the group of asthmatic patients and the control group for the *PAI-1* gene’s 4G/5G polymorphism (Lampalo et al., 2018). As a result, we initially discovered that in allergic individuals, the 4G/5G polymorphism of the *PAI-1* gene related to cough performance rather than asthma. Future mechanistic components of the study might need to pay greater attention to the correlation between a cough and this gene in allergy patients rather than only asthma patients.

Nevertheless, there are some limitations in our study. Because of the small sample size, we had to be cautious when interpreting our findings. The small sample size of some group may cause lower power value. Since the primary result is a positive result, it is not affected by low power values. However, negative results may have some possibility of committing type II errors. In addition, it is essential to conduct future multi-center, large-scale, long-dimensional research to clarify the function of *PAI-1* 4G/5G polymorphisms in allergic patients with cough. Furthermore, we have not looked at how allergy individuals’ *PAI-1* 4G/5G polymorphisms relate to *PAI-1*. For the treatment and prevention of cough and other *PAI-1*-associated disorders, additional research examining the mechanisms of PAI-1 activity and regulation may result in the creation of a unique prognostic factor and therapeutic target. The strength of this study lies in the cross-sectional study of multiple loci polymorphisms in multiple allergic diseases. The rich variety of allergic diseases points to the direction for subsequent studies.

## 5 Conclusion

In conclusion, we discovered that allergic individuals with cough rather than asthma had a distinct distribution of *PAI-1* rs1799762 genotypes. Allergic cough patients were more likely to have the 4G4G and 5G5G genotypes, whereas healthy people were more likely to have the 4G5G genotype.

## Data Availability

The original contributions presented in the study are included in the article/Supplementary Materials, further inquiries can be directed to the corresponding authors.
